# One dimensional detection for radio signals based on SpecUNeSt-MLP

**DOI:** 10.1371/journal.pone.0359267

**Published:** 2026-10-13

**Authors:** Chongxiao Qu, Jiali Cai, Guohua Zhang, Zhizhou Liao, Xinyu Chen, Baoqing Zeng

**Affiliations:** 1 University of Electronic Science and Technology of China, Chengdu, Sichuan, China; 2 52nd Research Institute of China Electronics Technology Group Corporation, Hangzhou, Zhejiang, China; 3 China Nanhu Academy of Electronics and Information Technology, Jiaxing, Zhejiang, China; Mae Fah Luang University, THAILAND

## Abstract

Detecting and precisely localizing multiple radio signals in wideband power spectral density (PSD) vectors under low signal-to-noise ratio (SNR) conditions remains a fundamental challenge for spectrum monitoring and cognitive radio. Traditional energy detection and threshold-based methods suffer from the SNR-wall effect and poor performance in dense, non-cooperative environments. While deep-learning approaches have advanced the field, most treat spectrum sensing as a binary classification task, discarding the fine-grained frequency-bin information needed for accurate signal boundary and parameter estimation. In this paper, we propose **SpecUNeSt-MLP**, an one-dimensional semantic segmentation framework built upon the U-Net encoder-decoder architecture. The model directly predicts per-frequency-bin occupancy masks from normalized PSD vectors, thereby simultaneously achieving signal detection and parameter estimation (center frequency and bandwidth). Three key enhancements are introduced: (1) a 1D-adapted Split-Attention mechanism in the encoder that dynamically recalibrates cross-channel features to enhance weak-signal extraction under heavy noise; (2) a Bidirectional Rolling MLP (RMLP) module in the bottleneck that efficiently captures long-range spectral dependencies with linear complexity; and (3) Feature Incentive Blocks that refine feature representations through lightweight channel projection. Extensive experiments on 67,200 synthetic broadband PSDs and 820 real-world UAV PSD samples demonstrate the effectiveness of SpecUNeSt-MLP. Across all tested SNR levels (from −4 dB to 8 dB), SpecUNeSt-MLP consistently outperforms LAD, FCN, SS-CNN, and SS-LSTM in terms of detection probability, false alarm rate, and average localization error. Compared with the recent RMP baseline, SpecUNeSt-MLP achieves better performance under low-SNR conditions while maintaining comparable performance at higher SNR levels, with only one-sixth of the parameter count of RMP. These results demonstrate that SpecUNeSt-MLP provides an effective and computationally efficient solution for fine-grained spectrum sensing in complex electromagnetic environments.

## 1. Introduction

The rapid growth of 5G/6G networks and Internet-of-Things devices has made the electromagnetic spectrum increasingly scarce [1]. Cognitive radio and dynamic spectrum access address this issue by enabling opportunistic spectrum utilization [2]. However, in non-cooperative scenarios such as spectrum monitoring and signal reconnaissance, receivers lack prior knowledge of signal parameters, making blind signal detection and localization essential. Moreover, modern spectrum sensing requires not only signal presence detection but also accurate estimation of signal number, center frequency, and bandwidth [3].

Traditional methods, including energy detection and cyclostationary feature detection, rely on handcrafted features and threshold selection [2,4]. Although computationally efficient, they suffer from noise uncertainty, the “SNR-wall” problem, and limited performance in dense spectral environments. The LAD method [5] improves localization capability but remains sensitive to threshold settings.

Deep learning has recently enabled end-to-end feature learning for spectrum sensing. CNN- and RNN-based approaches initially treated sensing as a classification task [6], but they neglect frequency-bin localization information. Recent studies therefore formulate signal detection as a 1D semantic segmentation problem [7,8], predicting occupancy masks over PSD bins to directly locate signal boundaries and estimate parameters. However, spectrum sensing based on one-dimensional power spectral signals still faces two critical challenges:

- **Long-range dependencies:** Wideband signals often contain multiple distributed narrowband components whose spectral relationships span a large frequency range. Conventional CNN-based models, such as SS-CNN [9], are constrained by limited receptive fields, while LSTM-based models, such as SS-LSTM [10], suffer from sequential processing limitations when modeling long-range spectral interactions. Transformer-based alternatives provide global attention mechanisms but introduce quadratic computational complexity with respect to sequence length, which restricts their application in real-time spectrum monitoring [11].
- **Noise robustness:** Weak signal features must be reliably distinguished from strong, fluctuating background noise; conventional convolutional blocks lack mechanisms for dynamic cross-channel recalibration under low SNR.

Recently, Rolling Multilayer Perceptron (Rolling-MLP) was proposed for efficient long-range feature modeling in two-dimensional medical image segmentation tasks [12,13]. By progressively shifting local windows, Rolling-MLP achieves global information interaction with linear computational complexity. However, directly applying the original Rolling-MLP paradigm to one-dimensional spectrum detection remains challenging because spectral signals have unique frequency-domain characteristics that differ from image structures. In particular, the original design lacks explicit mechanisms for exploiting bidirectional frequency dependencies and enhancing weak signal representations under noisy environments.

Inspired by the efficient long-range dependency modeling capability of Rolling-MLP, we adapt and extend this paradigm for one-dimensional PSD-based spectrum detection.Specifically, we propose SpecUNeSt-MLP, a lightweight 1D U-Net-style framework that incorporates the Rolling-MLP paradigm for long-range dependency modeling and Split-Attention mechanisms for adaptive feature fusion, specifically tailored for PSD-based multi-signal detection.Our main contributions are threefold:

- We formulate multi-signal detection and localization as a 1D semantic segmentation problem. The network outputs a per-bin occupancy mask that simultaneously enables detection and direct estimation of center frequency and bandwidth via the metric *E*_avg_.
- We introduce a 1D-adapted Split-Attention mechanism into the encoder that performs dynamic feature grouping and cross-group attention, substantially improving weak-signal discrimination in heavy noise.
- We design a Bidirectional Rolling MLP (RMLP) module for the bottleneck, augmented with Feature Incentive Blocks, that captures long-range spectral dependencies in both directions with only linear complexity while preserving local context through depthwise separable convolutions.

Overall, SpecUNeSt-MLP integrates these components into a unified 1D framework, providing an effective and lightweight solution for fine-grained spectrum detection and localization under challenging SNR conditions. Experiments on synthetic data and 820 real-world PSD samples show that SpecUNeSt-MLP outperforms FCN, SS-CNN, SS-LSTM, and LAD across all tested SNRs down to −4 dB. With fewer than one-sixth of RMP’s parameters, it achieves comparable or better low-SNR performance.

## 2. Related work

### 2.1. Classical spectrum sensing methods

Classical spectrum sensing (SS) techniques mainly include blind detection, feature-based detection, and matched filter-based approaches. Energy detection (ED) estimates average signal power and compares it with a threshold, offering low computational complexity but suffering from noise uncertainty and the resulting “SNR wall” in low-SNR regimes [2,4,14]. Cooperative spectrum sensing (CSS) improves detection reliability by aggregating information from multiple SUs [15,16]. Li et al. [15] proposed a CNN-LSTM CSS framework combining spatial and temporal features, while Janu et al. [16] introduced hierarchical LSTM-based cooperative sensing. However, recurrent architectures generally incur higher computational complexity and inference latency [17].

Eigenvalue-based detectors (EVD), such as MED, exploit covariance matrix properties and can outperform ED under low-SNR and high-noise conditions [18–20]. Nevertheless, they remain sensitive to noise-modeling errors and involve considerable matrix-operation overhead [21]. Cyclostationary detection improves noise robustness by exploiting periodic signal correlations, but requires intensive computation and prior knowledge of cyclic frequencies [5,22]. Matched filter-based methods achieve optimal detection with known signal models but require accurate prior information about PU parameters [23]. Overall, classical methods have limited adaptability to dynamic RF environments [23,24].

### 2.2. Deep learning-driven spectrum sensing

DL-based SS enables end-to-end feature learning from raw signals and reduces dependence on handcrafted features [9,16]. Existing approaches mainly employ CNNs, RNN/LSTMs, and hybrid architectures.

#### 2.2.1. CNN and RNN/LSTM-based architectures.

Early methods used 2D CNNs with spectrogram representations, but this introduces unnecessary computational overhead for inherently 1D spectral signals [6,9,25,26]. Therefore, 1D CNNs have emerged as a lightweight alternative for direct spectral feature extraction [27,28]. Mondal et al. [28] proposed a hybrid CNN-recurrent framework that jointly exploits spatial and temporal features for low-SNR sensing.

LSTMs are widely adopted for modeling temporal dependencies in sequential spectral data [29,30]. Soni et al. [29] developed an LSTM-based SS scheme that improves low-SNR detection by learning temporal correlations. Xing et al. [10] combined self-attention, 1DCNN, and BiLSTM to enhance global context modeling, but at the cost of increased inference latency. Serghini et al. [9] proposed a hierarchical 1DCNN-BiLSTM-MLP framework for cooperative sensing. Hybrid CNN-LSTM/GRU models further combine local and temporal feature extraction [16,30]. However, most existing models focus on binary occupancy classification rather than fine-grained spectral segmentation [31].

#### 2.2.2. Transformer and attention-based architectures.

Transformers have been introduced into spectrum sensing because self-attention can capture long-range dependencies [28,32]. Efficient Transformer and CNN–Transformer architectures have been investigated to reduce computational overhead [27]. However, lightweight Transformer applications to one-dimensional spectrum sensing and blind radio signal localization remain limited. Xing et al. [10] demonstrated improved global context extraction using self-attention, but with increased inference latency.

### 2.3. Lightweight neural architectures for RF signal detection

Lightweight neural architectures aim to reduce the computational and memory costs of DL-based RF detection, facilitating deployment on resource-constrained devices [3,17]. Serghini et al. [9] demonstrated the efficiency of 1D CNNs for edge-based CSS. Depthwise separable convolutions, channel attention, and feature reuse have also achieved favorable accuracy-efficiency trade-offs in RF signal processing [27]. Compact Transformer and CNN–Transformer structures further reduce parameter redundancy and inference latency [23]. Nevertheless, blind radio signal detection and localization in non-cooperative environments remain relatively underexplored, particularly under low-SNR conditions.

### 2.4. MLP-driven architectures for feature learning and segmentation

MLPs have reemerged as a cost-effective approach for global feature learning, offering competitive performance with relatively low complexity [11,33]. MLP-Mixer separates token and channel mixing for global feature interaction, while CycleMLP and AS-MLP introduce shifting operations to improve local feature extraction [11,34,35].

MLP-based segmentation models further demonstrate their ability to capture global dependencies efficiently. UNeXt, a lightweight CNN-MLP hybrid, achieved strong segmentation performance with only 1.47M parameters [34]. Rolling-Unet introduced Rolling-MLP modules and their orthogonal variants to model long-distance dependencies while combining local and global features [33]. RMMLP further employed dynamic matrix decomposition and rolling operations for adaptive feature mixing [36]. These studies indicate that MLP-based feature mixing can provide an efficient alternative to Transformer-based global modeling.

## 3. Method

In this section, we first formulate the one-dimensional radio signal detection problem as a semantic segmentation task on the power spectrum. We then present the overall architecture of the proposed SpecUNeSt-MLP framework, followed by detailed descriptions of its key components: the encoder with split attention and the rolling MLP bottleneck.

### 3.1. Problem definition

We consider a non-cooperative wideband spectrum sensing scenario where a receiver captures RF data at a sampling rate $F_{s}$ over a duration *T*. Within the observed wideband, multiple heterogeneous transmitters may be emitting signals simultaneously. The received discrete-time signal *r*(*n*) can be modeled as the superposition of $N_{sig}$ unknown signals $s_{i}(n)$ and additive noise *w*(*n*):


$$\begin{array}{c} r(n)=\sum_{i=1}^{N_{sig}}s_{i}(n)+w(n),\;n=0,1,...,N-1 \end{array}$$ (1)

where *w*(*n*) is assumed to be Additive White Gaussian Noise (AWGN). The parameters of each signal $s_{i}(n)$, including its modulation type, carrier frequency $f_{i}$, bandwidth $B_{i}$, and power, are unknown and vary dynamically.

Unlike traditional binary detection that outputs a single decision bit for the entire band, our goal is **Multi-Signal Parameter Estimation**. We aim to determine:

1. The number of active signals $N_{sig}$.
2. For each detected signal *i*, its precise frequency boundaries: the lower bound $f_{lower}^{i}$ and the upper bound $f_{upper}^{i}$ (from which the center frequency ${\hat{f}}_{i}$ and bandwidth ${\hat{B}}_{i}$ can be derived).

To achieve fine-grained detection, we transform this problem into a **binary segmentation task** in the frequency domain.

Let $𝐗\in {\mathbb{R}}^{K}$ denote the Power Spectral Density (PSD) vector of length *K* (obtained via FFT or Welch’s method). We define a binary mask (or label map) $𝐘\in \{0,1{\}}^{K}$, where:


$$\begin{array}{c} Y(k)=\left\{ \begin{array}{cc} 1 & \text{if the }k\text{-th frequency bin contains signal energy},\\ 0 & \text{if the }k\text{-th frequency bin contains only noise}. \end{array}\right. \end{array}$$ (2)

The multi-signal detection problem is then reformulated as a pixel-wise classification problem: we aim to learn a mapping function $f_{\Theta }(\cdot )$ (parameterized by our neural network Θ) that predicts the mask $\hat{𝐘}$ from the input spectrum 𝐗:


$$\begin{array}{c} \hat{𝐘}=f_{\Theta }(𝐗) \end{array}$$ (3)

Once the binary mask $\hat{𝐘}$ is obtained, the physical parameters are extracted algorithmically. Each continuous segment of “1"s in the mask corresponds to a detected signal. For the *i*-th continuous segment starting at index $I_{start}^{i}$ and ending at $I_{end}^{i}$, the estimated parameters are:


$$\begin{array}{c} {\hat{f}}_{lower}^{i}=\frac{F_{s}}{K}\cdot I_{start}^{i},\;{\hat{f}}_{upper}^{i}=\frac{F_{s}}{K}\cdot I_{end}^{i} \end{array}$$ (4)

$$\begin{array}{c} {\hat{f}}_{i}=\frac{1}{2}({\hat{f}}_{lower}^{i}+{\hat{f}}_{upper}^{i}),\;{\hat{B}}_{i}={\hat{f}}_{upper}^{i}-{\hat{f}}_{lower}^{i} \end{array}$$ (5)

where $F_{s}/K$ is the frequency resolution. This formulation explicitly decouples the “feature learning” (done by the CNN) from the “parameter estimation” (done by the mask decoding), providing a mathematically rigorous foundation for our deep learning approach.

### 3.2. Overall architecture

SpecUNeSt-MLP is a 1D deep learning framework tailored for PSD-based signal detection, built on the U-Net encoder-decoder architecture with three key enhancements: split attention mechanism, rolling MLP (RMLP) module, and feature incentive blocks. The overall pipeline is illustrated in Fig 1, consisting of four main stages:

**Fig 1:**
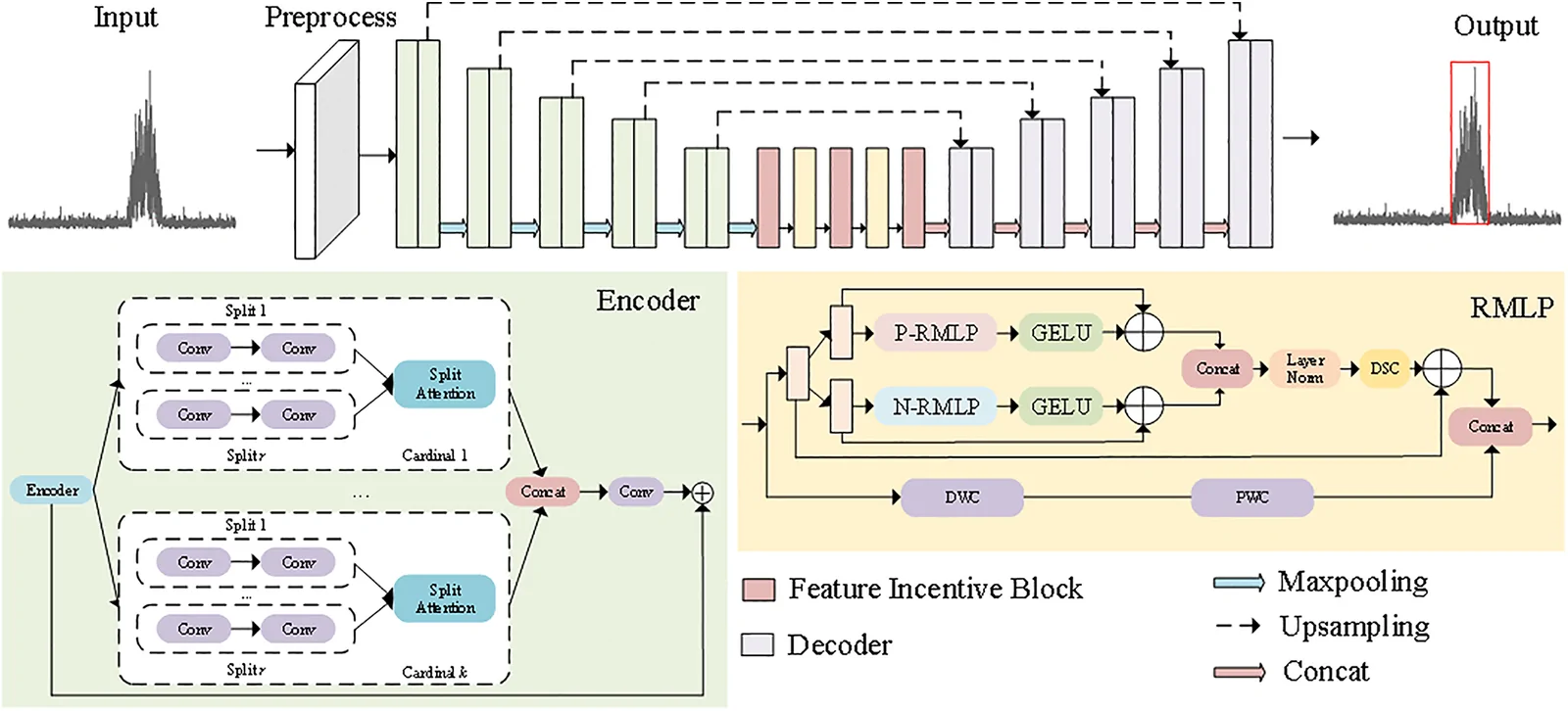
Overall architecture of the proposed SpecUNeSt-MLP. The encoder incorporates split attention blocks, the bottleneck uses rolling MLP modules, and skip connections preserve spatial details.

- Input Preprocessing: The raw PSD vector (1×L) is first subjected to logarithmic transformation and Z-score normalization. Subsequently, a three-layer convolutional stem extracts shallow spectral features and projects the input from 1×L to a $C_{0}\times L$ feature representation, providing a stable and informative embedding for the subsequent encoder.
- Encoder: At the *l*-th encoder stage, the input feature map $\left(C_{l-1}\times L_{l-1}\right)$ is first processed by a split‑attention module to extract multi‑scale spectral features and enhance cross‑channel interactions, followed by a convolutional block that projects the feature channels from $C_{l-1}$ to $C_{l}$. Finally, max‑pooling performs spatial down‑sampling, producing an output feature map of size $C_{l}\times L_{l}$.
- Bottleneck: The bottleneck is composed of two Bidirectional RMLP layers and three Feature Incentive Blocks (FIBs). Each RMLP layer captures long-range dependencies along the frequency axis with linear computational complexity and incorporates depthwise separable convolution (DSC) to preserve local contextual information. The FIBs perform lightweight channel projection through channel compression and restoration, improving feature representation while reducing computational overhead. The bottleneck preserves both the sequence length and channel dimension, ensuring that the input and output feature maps have identical dimensions and enabling seamless interaction with the encoder and decoder.
- Decoder: Each decoder stage first upsamples the input feature map using nn.Upsample and concatenates it with the corresponding encoder feature map before the max-pooling operation via a skip connection. The fused features are then processed by two consecutive convolutional blocks for feature refinement. Each decoder stage restores the feature map to the same spectral resolution and channel dimension as the input to the corresponding encoder stage.

This design synergistically integrates a 1D split-attention encoder, a bidirectional Rolling MLP bottleneck, and U-Net skip connections. To address the high computational overhead and long inference time associated with conventional Transformer self-attention, the 1D split-attention mechanism enhances subgroup features and strengthens cross-channel interactions through global context aggregation and dynamic weighting. Meanwhile, the bidirectional Rolling MLP employs parameter-free rolling shifts to establish linear long-distance receptive fields across channels. Consequently, compared with conventional MLP- and Transformer-based models, SpecUNeSt-MLP maintains high detection and localization accuracy while reducing computational overhead, thereby achieving a more favorable balance between accuracy and computational efficiency.

### 3.3. Encoder with split attention mechanism

One of the primary challenges in spectrum signal detection is to extract weak target features from complex background noise. These signals typically exhibit coupled multi-frequency components, strong correlations between local time-frequency structures and global context, and significant spectral overlap between the signal of interest and noise. Conventional U-Net encoders rely on standard ResNet blocks as their basic building blocks, which mitigate gradient vanishing through residual connections. However, these blocks are limited in modeling cross-frequency dependencies and capturing fine-grained feature variations. Standard convolutional operations primarily focus on local frequency neighborhoods, lacking mechanisms for dynamic weighting and global integration across frequency bands, which leads to inadequate representation of weak signals.

ResNeSt (Split-Attention Networks) introduces an effective Split-Attention mechanism that enables cross-group attention interactions while preserving the modularity and computational efficiency of ResNet, achieving superior performance in image classification and visual tasks [37]. Given the structural analogy between 1D spectrum data and 2D images—both benefit from dimension grouping, local feature extraction, and global fusion—this work adapts the Split-Attention mechanism to one-dimensional spectrum processing and employs it as the core building block of the U-Net encoder. The adapted encoder structure is illustrated in Fig 1. consisting of three main components: (1) feature grouping and local extraction, (2) split-attention computation, and (3) residual shortcut connection. The design details of each component are described below.

#### 3.3.1. Feature grouping and local feature extraction.

Let the input feature map be $X\in {\mathbb{R}}^{L\times C}$, where *L* denotes the length of the frequency dimension (e.g., the number of FFT points) and *C* the number of channels. To effectively capture the multi-scale frequency characteristics of the feature map, two hyperparameters are introduced: (a) Cardinality *K*: it partitions the input channels into K cardinal groups, with each group consisting of channels that exhibit similar frequency responses; (b) Radix *R*: it further splits each cardinal group into R subgroups (splits) to implement finer-grained local feature modeling. The total number of subgroups is thus G=K×R. For each subgroup, local features are extracted via a sequential combination of 1D convolutions:

- A 1D pointwise convolution (kernel size 1×1) followed by Batch Normalization (BN) and ReLU, which compresses the channel dimension to $C^{\prime }/(KR)$ (where $C^{\prime }$ is the intermediate channel count) and introduces channel-wise nonlinearity.
- A 1D convolution with kernel size 3×1 followed by BN and ReLU, which captures local correlations along the frequency axis.

The output of the *i*-th subgroup is denoted as $U_{i}\in {\mathbb{R}}^{L\times C^{\prime }/K}$, i=1,...,G.

#### 3.3.2. Split-attention mechanism.

The split-attention mechanism enhances subgroup features through global context aggregation and dynamic weighting, as shown in Fig 2. It comprises three steps:

**Fig 2:**
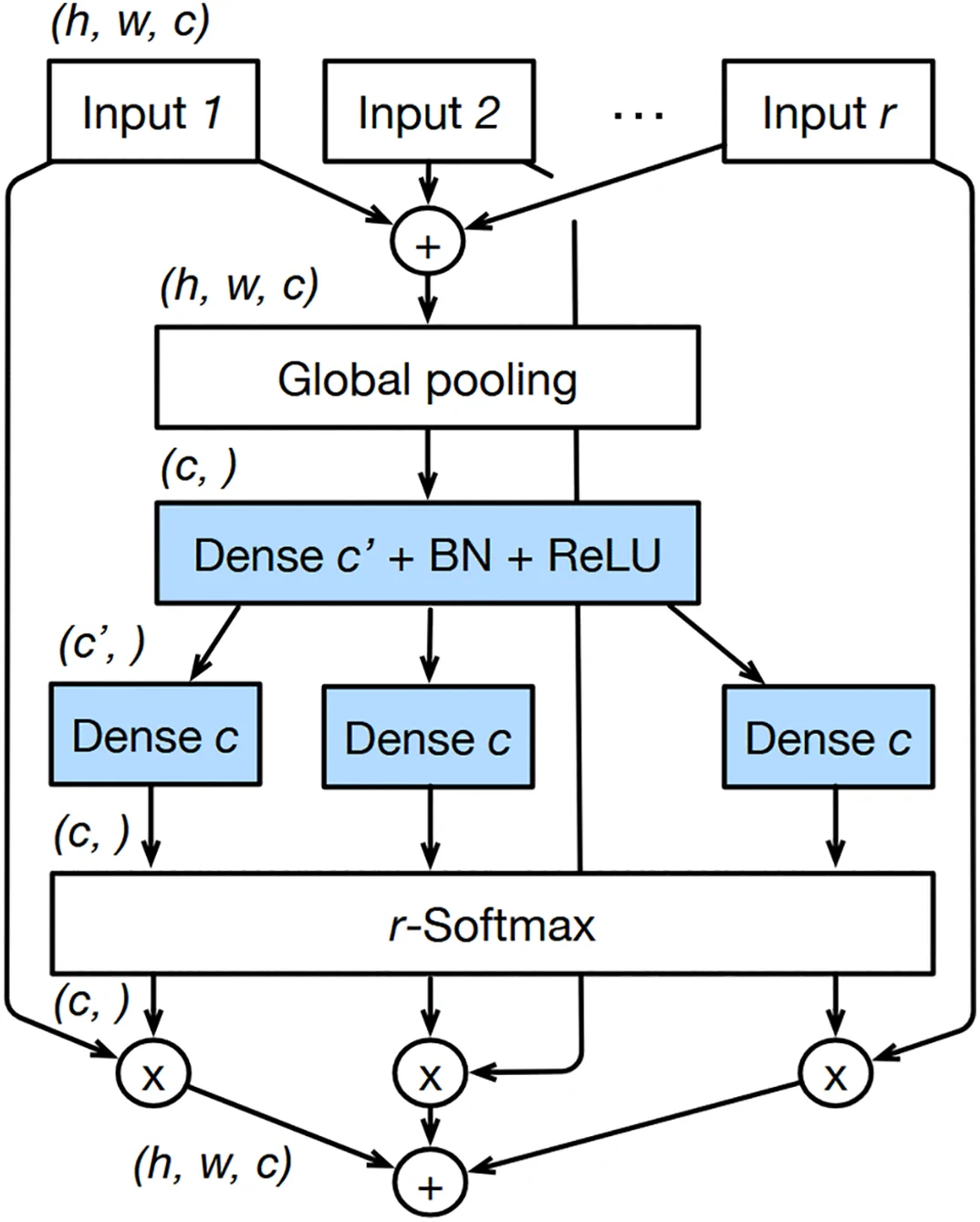
Split-Attention within a cardinal group.

**Cardinal Group Aggregation** For the *k*-th cardinal group (k=1,...,K), the *R* corresponding subgroup features are summed element-wise:


$$\begin{array}{c} {\hat{U}}^{k}=\sum_{j=R(k-1)+1}^{Rk}U_{j}, \end{array}$$ (6)

yielding ${\hat{U}}^{k}\in {\mathbb{R}}^{L\times C^{\prime }/K}$.

**Global Context Extraction** Global frequency context is obtained via 1D global average pooling (GAP) on each aggregated cardinal feature. For the *c*-th channel of the *k*-th cardinal group, the context descriptor is:


$$\begin{array}{c} s_{c}^{k}=\frac{1}{L}\sum_{i=1}^{L}{\hat{U}}_{c}^{k}(i). \end{array}$$ (7)

**Attention Weight Computation and Feature Fusion** Attention weights are learned through a two-layer fully connected (FC) bottleneck: a): The first FC layer maps the context vector $s^{k}\in {\mathbb{R}}^{C^{\prime }/K}$ to a lower-dimensional space ${\mathbb{R}}^{C^{\prime \prime }}$ ($C^{\prime \prime }=C^{\prime }/r$, where *r* is the reduction ratio), followed by BN and ReLU; b): The second FC layer expands the representation to ${\mathbb{R}}^{R\times C^{\prime }/K}$, producing per-channel weights for the *R* splits.

Softmax is applied channel-wise to normalize the weights:


$$\begin{array}{c} a_{i}^{k}(c)=\frac{\exp({𝒢}_{i}^{c}(s^{k}))}{\sum_{j=1}^{R}\exp({𝒢}_{j}^{c}(s^{k}))}, \end{array}$$ (8)

where ${𝒢}_{i}^{c}(\cdot )$ denotes the mapping for the *i*-th split and *c*-th channel.

The enhanced feature for the *k*-th cardinal group is obtained by weighted summation:


$$\begin{array}{c} V_{c}^{k}=\sum_{i=1}^{R}a_{i}^{k}(c)\cdot U_{R(k-1)+i,c}. \end{array}$$ (9)

Finally, the *K* enhanced cardinal features are concatenated along the channel dimension to form $V=\text{Concat}\{V^{1},...,V^{K}\}\in {\mathbb{R}}^{L\times C^{\prime }}$.

#### 3.3.3. Residual connection and dimension alignment.

To prevent gradient degradation, a residual shortcut is employed:

- If *X* and *V* have matching dimensions, the output is *Y* = *V* + *X*.
- If channel numbers or spatial (frequency) dimensions differ (e.g., due to downsampling), a projection transformation *T*(*X*) implemented by a 1D strided convolution followed by BN is applied: *Y* = *V* + *T*(*X*).

The block output *Y* (possibly with reduced frequency length $L^{\prime }$ after downsampling) is fed to the next layer.

### 3.4. Bottleneck: rolling MLP module

To address the limited ability of convolution-based operations to capture dependencies between distant frequency bins, we integrate a Rolling MLP module into the bottleneck of SpecUNeSt. The convolutional encoder effectively extracts local spectral structures, while the split-attention mechanism enhances subgroup features and cross-channel interactions. The bottleneck features contain high-level spectral semantics and have a reduced sequence length, making this stage suitable for efficient long-range feature interactions without altering the original encoder architecture or U-Net skip connections. Accordingly, we adapt and extend the Rolling MLP mechanism originally proposed for 2D medical image segmentation [12]. It employs parameter-free rolling shifts to establish linear long-distance receptive fields across channels, which are mapped to the frequency dimension for one-dimensional spectral signals. In this way, the Rolling MLP complements the convolutional components, allowing the network to jointly model local spectral patterns, cross-channel interactions, and long-range frequency dependencies.

#### 3.4.1. Rolling MLP (R-MLP) module.

The basic R-MLP module is designed to capture linear long-range dependencies along a single direction of the input sequence. Given an input feature map $𝐗\in {\mathbb{R}}^{C\times L}$, where *C* is the number of channels and *L* is the sequence length (spectral dimension), the rolling operation is applied channel-wise along the length axis. For each channel c∈[1,C], the feature vector ${𝐱}_{c}\in {\mathbb{R}}^{L}$ is cyclically shifted by an offset proportional to the channel index: $k_{c}=c\cdot k_{0}$, where *k*_0_ is a fixed base shift step (positive for forward direction), as shown in Fig 3. The shifted vector is then cropped to preserve length *L*, ensuring alignment across channels. This parameter-free rolling shift enables diverse long-range interactions without expanding the receptive field quadratically. The shifted features are concatenated along the channel dimension to form a mixed token sequence of length C·L, followed by a linear projection to restore the original channel dimension:


$$\begin{array}{c} 𝐘=\text{GELU}({𝐖}_{1}\cdot \text{Concat}(\{\text{Rolling}({𝐱}_{c},k_{c}){\}}_{c=1}^{C})+𝐛), \end{array}$$ (10)

where ${𝐖}_{1}\in {\mathbb{R}}^{C\times C}$ are learnable weights, and a residual connection is typically added: 𝐘←𝐘+𝐗. This formulation achieves near-global linear dependency modeling with complexity *O*(*C*^2^*L*), comparable to pointwise convolution yet far more effective for long spectral correlations.

**Fig 3:**
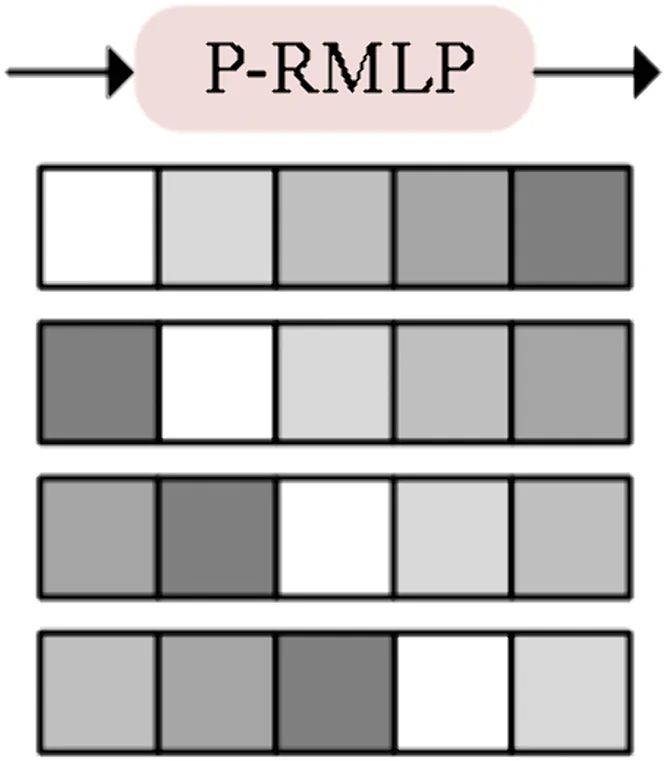
Rolling MLP (RMLP) module structure.

#### 3.4.2 Bidirectional RMLP Module.

Spectral signals often exhibit bidirectional contextual relationships—for instance, low-frequency trends influencing high-frequency transients and vice versa. To capture both forward and backward long-range dependencies, we propose a Bidirectional RMLP module consisting of two parallel R-MLP branches: a positive-direction branch (P-RMLP, *k*_0_ > 0) and a negative-direction branch (N-RMLP, *k*_0_ < 0). Each branch processes the input independently:


$$\begin{array}{c} {𝐘}_{p}=\text{GELU}({\text{R-MLP}}_{+}(𝐗))+𝐗,\\ {𝐘}_{n}=\text{GELU}({\text{R-MLP}}_{-}(𝐗))+𝐗. \end{array}$$ (11)

The outputs are concatenated along the channel dimension ($𝐙={𝐘}_{p}⊕{𝐘}_{n}$), followed by Layer Normalization for stable training. To preserve local spectral details without additional global overhead, we incorporate two parallel local mixing paths: (1) a depthwise separable convolution (DSC) applied directly to 𝐙, and (2) a depthwise convolution (DWC) followed by pointwise convolution (PWC) on 𝐙. The final output is obtained by fusing these paths with a residual connection:


$$\begin{array}{c} 𝐎=\text{PWC}(\text{DWC}(𝐙))⊕\text{DSC}(𝐙). \end{array}$$ (12)

The overall architecture of the Bidirectional RMLP module is illustrated in Fig. 1. This design doubles the directional coverage compared to the unidirectional R-MLP while maintaining similar computational cost, making it particularly suitable as the bottleneck replacement in U-Net-style encoders for long spectral sequences.

#### 3.4.3. Feature incentive block.

In the bottleneck of the network, channel reduction followed by channel restoration is adopted to reduce computational cost while maintaining representation capability. Following [12], we employ a Feature Incentive Block to refine feature representations through channel projection. The block consists of sequential operations: a 1×1 convolution for channel compression (to *C*/2), batch normalization, ReLU activation, followed by another 1×1 convolution for channel expansion (back to *C*), and a final residual addition to the input. Formally:


$$\begin{array}{cc} {𝐗}^{\prime } & ={\text{Conv}}_{1\times 1}^{C\to C/2}(𝐗),\\ \;{𝐗}^{\prime \prime } & =\text{ReLU}(\text{BN}({𝐗}^{\prime })),\\ 𝐎 & ={\text{Conv}}_{1\times 1}^{C/2\to C}({𝐗}^{\prime \prime })+𝐗. \end{array}$$ (13)

This lightweight structure incentivizes the network to focus on salient spectral features during compression, mitigating information loss before feeding into long-range modeling modules such as Bidirectional RMLP. In our architecture, Feature Incentive Blocks are placed immediately before Bidirectional RMLP blocks in the bottleneck to ensure efficient deep feature representation.

## 4. Experiments

To comprehensively evaluate the effectiveness of the proposed SpecUNeSt-MLP framework for one-dimensional radio signal detection, we perform extensive experiments. The model is trained and validated on 67,200 synthetic broadband PSDs that closely emulate real-world electromagnetic environments. This section details the dataset construction, baseline methods, implementation details, evaluation metrics, quantitative results, ablation studies, complexity analysis, and qualitative visualizations, highlighting the superiority of SpecUNeSt-MLP, particularly in challenging low-SNR regimes.

### 4.1. Experimental setup

In this section, we describe the experimental configuration, including the baseline methods, evaluation metrics, and implementation details. All experiments are conducted under the same hardware and software environment to ensure fairness.

#### 4.1.1. Datasets.

We collected real-world RF signals from multiple drone models operating in different frequency bands, primarily the 2.4 GHz and 5.8 GHz bands, and obtained 820 wideband PSD samples. Each wideband RF capture was sampled at a rate of 100 MHz over a duration of 20 ms. Each capture contained 5–49 narrowband signal components, with durations ranging from 0.1 to 3 ms and bandwidths ranging from 1 to 10 MHz. Each PSD sample were computed using an 8192-point fast Fourier transform (FFT). The corresponding labels were independently annotated by two experienced RF signal processing experts and subsequently cross-checked to ensure annotation consistency. Samples with inconsistent annotations were further reviewed and reconciled through discussion to minimize human annotation errors.

The SNR of each real-world sample was estimated using a bounding-box-based energy-ratio method, defined as the ratio of the signal energy within the manually annotated bounding boxes to the background-noise energy outside them. The estimated SNRs ranged from 4.8 dB to 31.4 dB. Since the original real-world samples were predominantly high-SNR, they provided limited coverage of low-SNR conditions. We therefore added controlled additive white Gaussian noise (AWGN) to the original samples to evaluate the robustness of the proposed method across SNR levels ranging from −4 dB to 8 dB in 2 dB increments, while maintaining an approximately balanced number of samples at each SNR level. Finally, 400 noise-corrupted samples were used for training and 420 samples for testing, with 60 test samples available for each SNR level.

Given the limited size of the real-world dataset, which is insufficient to support the training of deep CNN models, we generated 67,200 simulated broadband power spectra for model training using a simulation framework implemented in MATLAB. The SNR settings of the synthetic dataset were consistent with those of the real-world dataset, with 9,600 samples generated for each SNR level.The modulation schemes employed in the simulation framework include 2ASK, BPSK, QPSK, 2FSK, and MSK, with root-raised cosine (RRC) filters used for pulse shaping except for 2FSK and MSK. The duration of each narrowband signal ranges from 0.1 ms to 3 ms, the carrier frequency spans 5–95 MHz, and the signal bandwidth varies between 1 MHz and 10 MHz. The number of narrowband signals within each wideband RF capture is randomly selected from the range [5, 49].Each generated RF capture was sampled at 100 MHz for 20 ms. The resulting 2,000,000-sample sequence was then segmented into 240 non-overlapping segments, each containing 8,192 samples, while the remaining samples were discarded. An 8,192-point FFT was subsequently applied to each segment to obtain 240 PSD samples. Therefore, by generating 40 wideband RF captures of 20 ms duration at each SNR level, a total of 67,200 simulated wideband PSD samples were obtained.

During model training, the 67,200 samples in the synthetic dataset were divided into 53,760 samples (80%) for training, 6,720 samples (10%) for validation, and 6,720 samples (10%) for testing. In addition, 420 real-world samples were included in the test set, resulting in a total of 7,140 test samples, with 1,020 samples at each SNR level. All experimental results reported in the remainder of this paper, including ablation studies, parameter analysis, and comparisons with existing methods, were obtained using this combined test set consisting of both synthetic and real-world samples.

#### 4.1.2. Baseline methods.

To validate the effectiveness of the proposed SpecUNeSt-MLP architecture, we compare it against the following state-of-the-art semantic segmentation and detection methods:

- **LAD (Likelihood Ratio Test with Adaptive Thresholding) [**5**]:** A traditional statistical signal processing method. It serves as a non-learning baseline to highlight the advantage of data-driven deep learning approaches in complex electromagnetic environments.
- **FCN (Fully Convolutional Network) [**7**]:** A method that transforms a wideband power spectral sequence into a one-dimensional image segmentation problem, utilizing an FCN to classify each spectral sampling point, thereby achieving carrier signal detection and localization.
- **SS-CNN [**9**]:** A one-dimensional convolutional neural network designed for spectrum sensing. By leveraging local receptive fields and weight sharing, it effectively extracts spectral features with relatively low computational cost and serves as a representative CNN-based baseline.
- **SS-LSTM [**10**]:** A spectrum sensing model based on Long Short-Term Memory (LSTM) networks, which exploits temporal dependencies in sequential spectrum data to distinguish signal and noise components. It serves as a representative recurrent neural network (RNN)-based approach for comparison.
- **RMP [**13**]:** A lightweight MLP-based segmentation network that employs Rolling MLP blocks to capture long-range dependencies while maintaining low computational complexity. It provides an efficient baseline for evaluating the effectiveness of MLP-based feature modeling in spectrum sensing.

#### 4.1.3. Evaluation metrics.

We employ four standard metrics to quantitatively assess the performance of wideband spectrum sensing, covering both pixel-level segmentation accuracy and signal parameter localization precision.

1. **Intersection over Union (IoU):** Also known as the Jaccard index, IoU measures the overlap between the predicted segmentation mask and the ground truth mask, which is a core metric for evaluating semantic segmentation performance. It is defined as the ratio of the intersection area to the union area of the two masks:

$$\begin{array}{c} \text{IoU}=\frac{TP}{TP+FP+FN} \end{array}$$ (14)

where *TP* (True Positive) represents correctly detected signal pixels, *FP* (False Positive) represents noise pixels incorrectly classified as signals, and *FN* (False Negative) represents signal pixels missed by the model. A higher IoU indicates better segmentation accuracy.

2. **Detection Probability (**$P_{d}$**):** The ratio of correctly detected signal segments to the total number of actual signal segments, reflecting the model’s ability to capture existing signals. Higher is better.

$$\begin{array}{c} P_{d}=\frac{TP}{TP+FN} \end{array}$$ (15)

3. **False Alarm Rate (**$P_{f}$**):** The ratio of noise segments incorrectly classified as signals to the total number of noise segments, measuring the model’s ability to avoid false detections. Lower is better.

$$\begin{array}{c} P_{f}=\frac{FP}{TN+FP} \end{array}$$ (16)

where *TN* (True Negative) represents correctly classified noise pixels.

4. **Average Localization Error (**$E_{avg}$**):** A comprehensive metric that jointly evaluates the accuracy of center frequency and bandwidth estimation for detected signals. It normalizes the absolute errors of center frequency and bandwidth by the ground-truth bandwidth, then averages over all detected signals:

$$\begin{array}{c} E_{avg}=\frac{1}{2N_{TP}}\sum_{i=1}^{N_{TP}}\left(\frac{\left|f_{i}-{\hat{f}}_{i}\right|}{B_{i}}+\frac{\left|B_{i}-{\hat{B}}_{i}\right|}{B_{i}}\right) \end{array}$$ (17)

where $f_{i}$ and $B_{i}$ are the ground-truth center frequency and bandwidth of the *i*-th signal, ${\hat{f}}_{i}$ and ${\hat{B}}_{i}$ are the corresponding predicted values, and $N_{TP}$ is the number of accurate predictions. A lower $E_{avg}$ indicates more accurate signal localization and parameter estimation.

#### 4.1.4. Implementation details.

**Network Hyperparameters.** Unless otherwise specified, the output channel dimension of each encoder stage is twice its input channel dimension. For the Split‑Attention module, the cardinality and radix are set to *K* = 2 and *R* = 2, respectively, and the intermediate channel dimension is defined as $c^{\prime }=2c$. The Preprocess module outputs feature maps with *C* = 16 channels. In the Bidirectional RMLP, the base shift step is set to *k*_0_ = 1. Other hyperparameters, such as the FFT size, network depth, and IoU threshold, are specified in the corresponding experimental sections according to the evaluation settings.

**Training Details.** The experiments are implemented using the PyTorch framework. All models are trained and evaluated on an NVIDIA Tesla V100 GPU with 32 GB of memory. To improve the reliability and statistical significance of the experimental results, each experiment is repeated five times using different random seeds. The reported performance metrics are presented as the mean values with standard deviations (mean ± std) calculated from the five independent runs. In the figures(Fig 4–8), the solid curves represent the mean performance, while the shaded regions around the curves indicate the corresponding standard deviations, reflecting the performance fluctuations caused by different random initializations.

**Fig 4:**
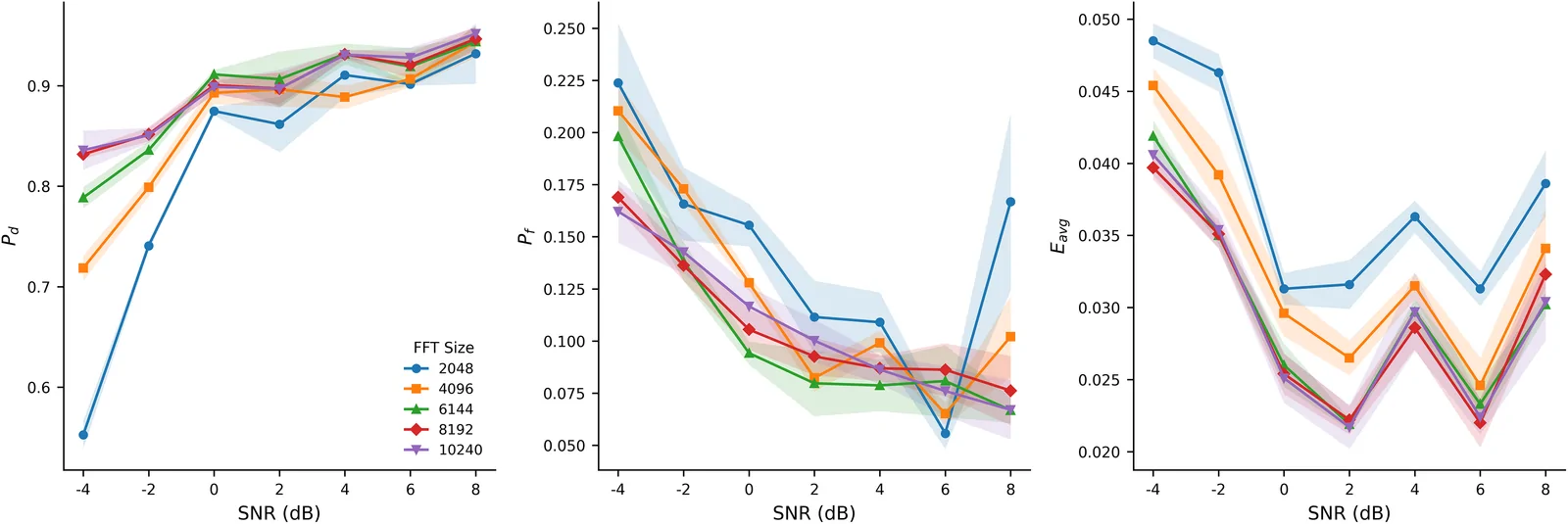
Performance comparison under different FFT points across SNR levels from −4 dB to 8 dB. (left) Detection Probability, (center) False Alarm Rate, (right) Average Localization Error.

**Fig 5:**
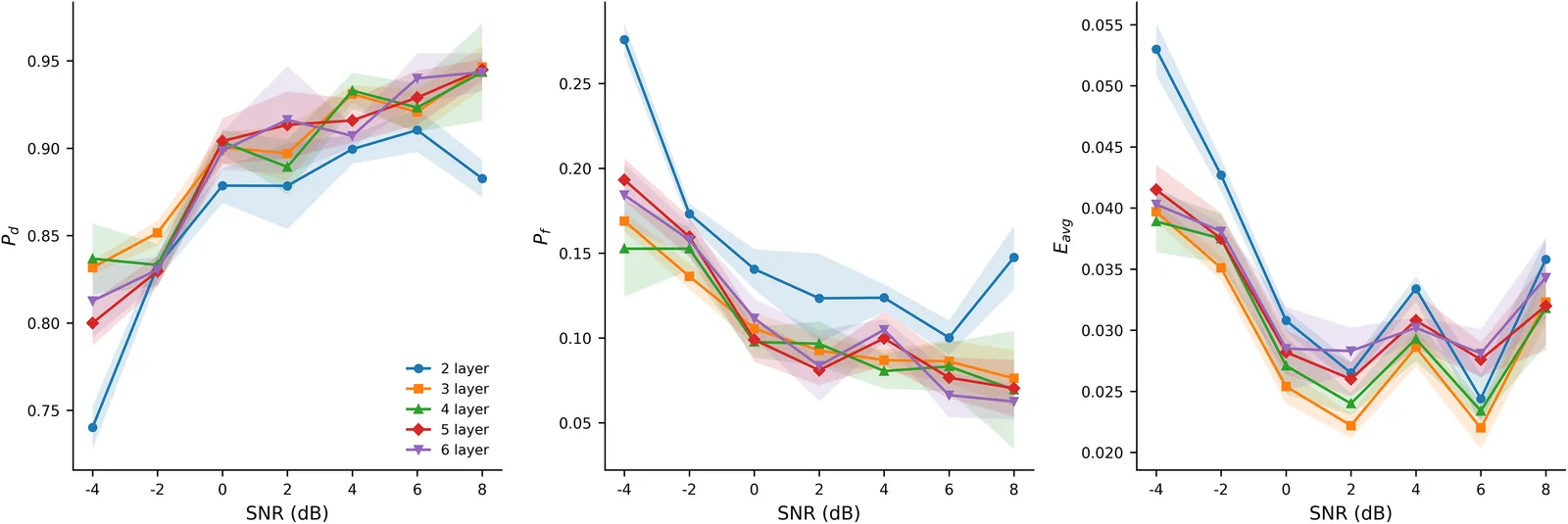
Performance comparison under different network depths across SNR levels from −4 dB to 8 dB. (left) Detection Probability, (center) False Alarm Rate, (right) Average Localization Error.

**Fig 6:**
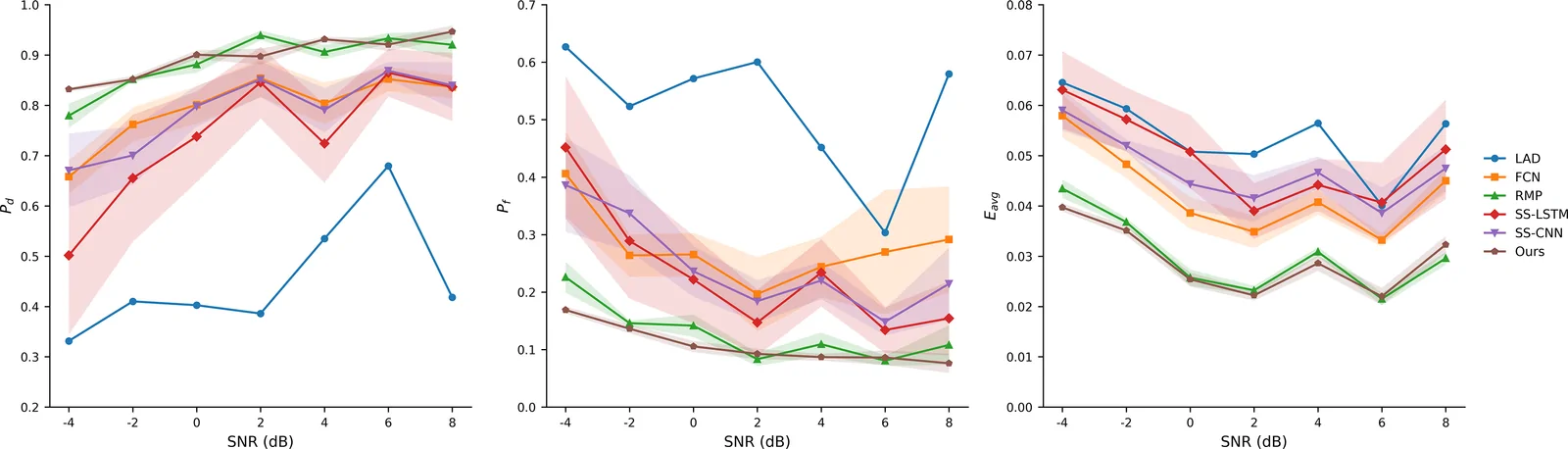
Comparison of SpecUNeSt-MLP with LAD, FCN, RMP, SS-LSTM, and SS-CNN under different SNR conditions. The curves show detection probability $P_{d}$ (left), false-alarm probability $P_{f}$(center), and average localization error $E_{avg}$(right). Note that the traditional LAD is a deterministic algorithm with no random initialization. Therefore, error bands are not displayed for the LAD curve.

**Fig 7:**
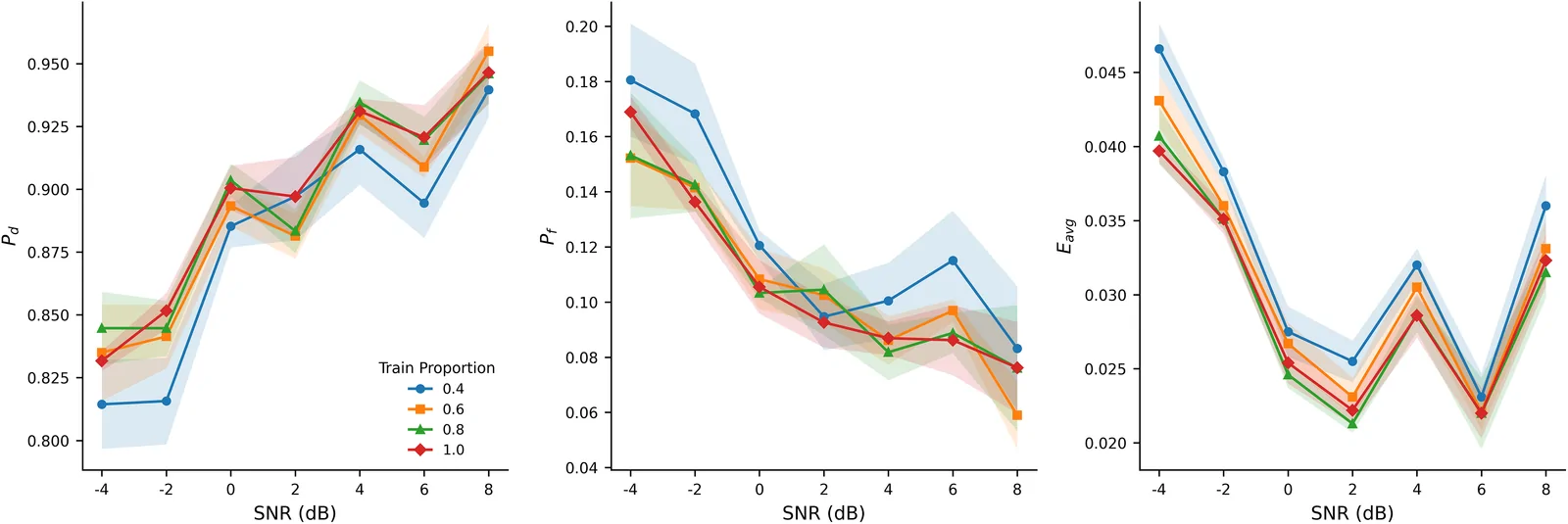
Performance under different training sample proportions across SNR levels from −4 dB to 8 dB. (left) Detection Probability, (center) False Alarm Rate, (right) Average Localization Error.

**Fig 8:**
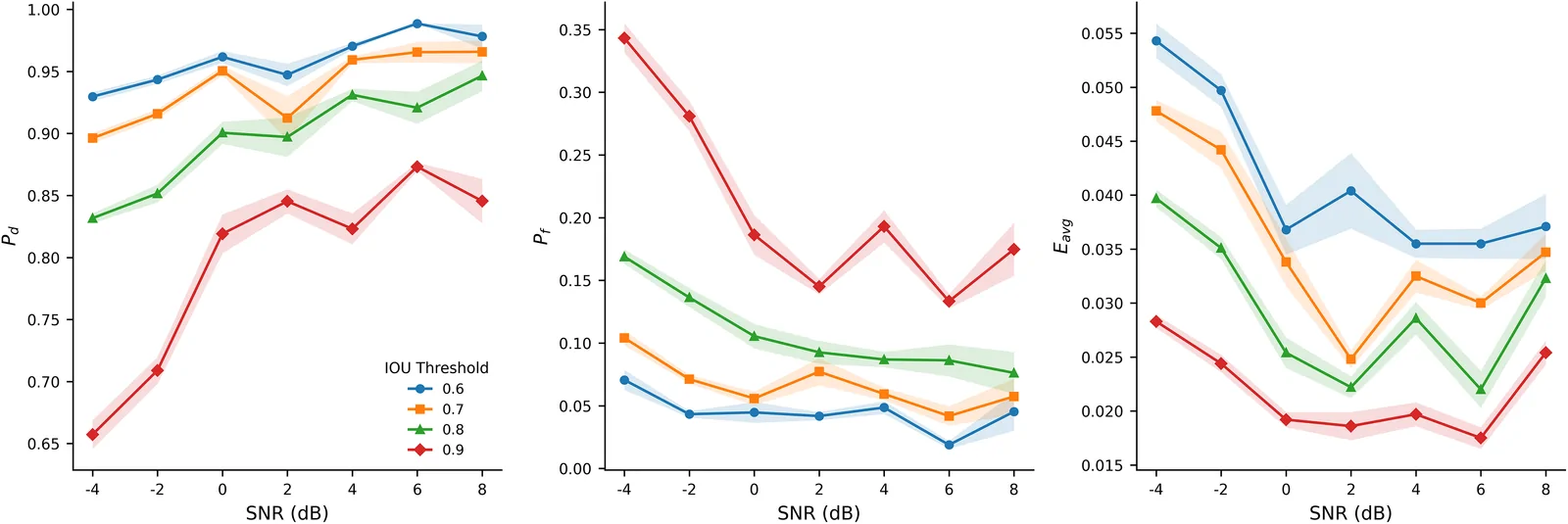
Performance comparison under different IoU thresholds across SNR levels from −4 dB to 8 dB. (left) Detection Probability, (center) False Alarm Rate, (right) Average Localization Error.

The Adam optimizer is adopted with an initial learning rate of $1\times 10^{-4}$ and a batch size of 16. The models are trained for 100 epochs using a step decay learning rate schedule, where the learning rate is multiplied by 0.5 every 50 epochs. The network input is the power spectral density generated from the raw IQ samples. For the spectrum sensing binary classification task, Binary Cross-Entropy (BCE) loss is employed as the optimization objective. Since the training data exhibit only moderate class imbalance (approximately 1:9 between signal and background bins), BCE provides stable optimization and satisfactory detection performance without requiring specialized loss functions such as Dice or Focal Loss. The BCE loss is defined as follows:


$$\begin{array}{c} {ℒ}_{\text{BCE}}=-\frac{1}{N}\sum_{i=1}^{N}\left[y_{i}\cdot \log({\hat{y}}_{i})+(1-y_{i})\cdot \log(1-{\hat{y}}_{i})\right] \end{array}$$ (18)

where *N* denotes the total number of pixels in the output prediction map, $y_{i}$ represents the ground-truth binary label (1 for signal pixels and 0 for noise pixels) of the *i*-th pixel, and ${\hat{y}}_{i}$ denotes the predicted probability score of the *i*-th pixel belonging to the signal class output by the model.

#### 4.1.5. Complexity analysis.

We analyze the model complexity in terms of parameter count and floating-point operations (FLOPs). Let *L* denote the spectral sequence length (FFT size), *C* denote the channel dimension.

**Parameter count.** The parameters originate from four sources:

**(1) 1D convolutions in the encoder and decoder.** For a 1D convolutional layer with kernel size $K_{i}$, input channels $C_{i-1}$, and output channels $C_{i}$, the parameter count is $K_{i}\cdot C_{i-1}\cdot C_{i}$. Let *I* denote the total number of 1D convolutional layers in the encoder and decoder.The total convolutional parameters are given by

$$\begin{array}{c} {𝒫}_{\text{conv}}=\sum_{i=1}^{I}K_{i}C_{i-1}C_{i}, \end{array}$$ (19)

which constitutes the major portion of the total parameter count. Note that this differs from the 2D case by using $K_{l}$ instead of $K_{l}^{2}$, as all convolutions in SpecUNeSt-MLP are 1D.

**(2) Split-Attention encoder (Sec. 3.3).** Let *J* denote the number of Split-Attention modules. For the *j*-th module, let $L_{j}$, $C_{j}$, $C_{j}^{mid}$, and *R* denote the spectral length, channel dimension, intermediate channel dimension, and radix, respectively. As shown in Fig 2, the learnable parameters are confined to two FC mappings, $C_{j}\to C_{j}^{mid}\to RC_{j}$. Their parameter count is

$$\begin{array}{c} {𝒫}_{SA}=\sum_{j=1}^{J}(R+1)C_{j}C_{j}^{mid}. \end{array}$$ (20)

According to Eqs. (6)–(9), split aggregation, global pooling, and weighted fusion require $O(RL_{j}C_{j})$ operations, while the two FC layers and radix softmax require $O(RC_{j}C_{j}^{mid}+RC_{j})$ operations. Since $RC_{j}$ is absorbed by $O(RL_{j}C_{j})$ for $L_{j}\geq 1$, the resulting complexity is


$$\begin{array}{c} {𝒞}_{SA}=O\;\left(\sum_{j=1}^{J}RC_{j}\left(L_{j}+C_{j}^{mid}\right)\right). \end{array}$$ (21)

The FC cost is not multiplied by $L_{j}$ because the FC layers operate on the globally pooled channel descriptor.

**(3) RMLP modules (Sec.3.4.2).** The bidirectional RMLP contains two independent linear projections (P-RMLP and N-RMLP). Per Eq. 7, each is a single linear layer with weight matrix ${𝐖}_{1}\in {\mathbb{R}}^{C\times C}$, contributing $C\times C=C^{2}$ parameters. The two branches therefore contribute 2*C*^2^. As described in Sec.3.4.2, the outputs are concatenated along the channel dimension to form $𝐙\in {\mathbb{R}}^{2C\times L}$, after which two parallel local mixing paths restore the channel count to *C*:
  - Depthwise separable convolution (DSC): depthwise 3×2C=6C parameters (one 3×1 kernel per input channel) and pointwise $(2C)\cdot C=2C^{2}$ parameters (mapping 2*C* back to *C*).
  - Depthwise convolution (DWC) + pointwise convolution (PWC): depthwise 3×C=3C parameters and pointwise $C\cdot C=C^{2}$ parameters (mapping from *C* to *C* channels).

Summing all terms, the bottleneck contributes ${𝒫}_{\text{btl}}\approx 5C^{2}+9C$.

**(4) Feature Incentive Blocks (Sec.3.4.3)**. Each block contains two 1×1 convolutions: the first compresses from *C* to *C*/2 ($C\cdot C/2=C^{2}/2$ parameters) and the second expands back to *C* ($C/2\cdot C=C^{2}/2$ parameters), totaling *C*^2^ parameters per block.

**Computational complexity.** The FLOPs are obtained by multiplying the parameter count at each layer by the output length *L*:

- Encoder/decoder convolutions: $O(\sum_{i=1}^{I}K_{i}C_{i-1}C_{i}L_{i})$.
- Split-Attention encoder: $O\;\left(\sum_{j=1}^{J}RC_{j}\left(L_{j}+C_{j}^{mid}\right)\right)$.
- Bidirectional RMLP linear projecitions: 2*C*^2^ *L* MACs.
- DSC and DWC + PWC in the bottleneck: 3*C*^2^ *L* + *O*(*C L*) MACs.
- Feature Incentive Blocks: *C*^2^ *L* MACs.

Taking all these into account, the overall computational complexity is


$$\begin{array}{c} O\;\left(\sum_{i=1}^{I}K_{i}C_{i-1}C_{i}L_{i}+\sum_{j=1}^{J}RC_{j}\left(L_{j}+C_{j}^{mid}\right)+C^{2}L+CL\right). \end{array}$$ (22)

For fixed channel dimensions and radix, the Split-Attention contribution grows linearly with the spectral length, while its two learnable FC projections contribute a length-independent term.

### 4.2. Impact of FFT points

To investigate the influence of FFT size on detection performance, we conducted experiments with five different FFT sizes: 2048, 4096, 6144, 8192, and 10240. For each FFT size, the same model architecture was used, while the corresponding spectral inputs were independently trained to accommodate the different input lengths. All other hyperparameters were kept consistent, using a 3-layer network, an IoU threshold of 0.8, and the full training set. The results are presented in Fig 4 with three subfigures showing Detection Probability ($P_{d}$), False Alarm Rate ($P_{f}$), and Average Localization Error ($E_{avg}$), respectively.

As shown in Fig 4(left), the detection probability $P_{d}$ increases consistently with the growth of FFT points. The most significant improvement occurs when the FFT size increases from 2048 to 8192. Further increasing the FFT size to 10240 yields only marginal gains.

From Fig 4(center) and (right), both the false alarm rate $P_{f}$ and the average localization error $E_{avg}$ decrease as the FFT resolution improves. Similar to the detection probability, only limited performance gains are observed when the FFT size exceeds 8192 points.

These results indicate that a larger FFT size provides higher frequency resolution, which is beneficial for distinguishing weak signals from noise and for more accurate localization of the signal center frequency and bandwidth. Therefore, increasing the FFT size can significantly improve the performance metrics of spectral detection. However, excessively large FFT sizes lead to substantially higher computational complexity and may also increase the difficulty of feature extraction due to the higher-dimensional and more detailed spectral representations. Moreover, the improvement in frequency resolution is accompanied by an increased signal observation time window, resulting in reduced time resolution and a greater risk of spectral overlap among multiple narrowband signals. These factors can limit the effective utilization of the additional spectral information and consequently lead to diminishing performance gains as the FFT size continues to increase. Therefore, the 8192-point FFT achieves a favorable trade-off between frequency resolution, detection performance, and computational efficiency, and is adopted as the default configuration in subsequent experiments.

### 4.3. Impact of network depth

To evaluate the effect of network depth on model performance, we compared five different depths: 2 layers, 3 layers, 4 layers, 5 layers and 6 layers, while keeping all other settings fixed (FFT size = 8192, IoU threshold = 0.8, full training set). The experimental results are shown in Fig 5, which consists of three subfigures corresponding to Detection Probability ($P_{d}$), False Alarm Rate ($P_{f}$), and Average Localization Error ($E_{avg}$), respectively.

As shown in Fig 5 (left), the detection probability ($P_{d}$) for all networks generally trends upward as the SNR increases. A significant improvement in detection probability is observed when the network depth increases from 2 to 3 layers; however, further increasing the depth to 4, 5, or 6 layers yields only marginal gains, with the detection performance of the 3–6 layers networks remaining very similar overall. Across most SNR conditions, the 3 layers network achieves a detection probability nearly equivalent to that of the 4, 5 and 6 layers networks; notably, at an SNR of −2, the 3 layers network significantly outperforms the others. Consequently, increasing the number of layers beyond this point offers very limited performance gains and fails to produce a significant leap in detection performance.

Fig 5(center) and (right) show similar trends for the false alarm rate and localization error. The performance of $P_{f}$ with 3 layers is similar to that with 4 layers, but decreases slightly slightly at high SNR; $E_{avg}$ reaches its minimum value at three layers. The 3-layer network has a parameter count of only 0.2M, whereas the 4-layer network increases to 0.7M, and the 5-layer and 6-layer networks reach 3M and 12M, respectively. This demonstrates that deep networks with four or more layers introduce significant parameter redundancy, substantially increasing computational overhead and deployment costs without yielding effective performance gains; instead, they are prone to issues such as vanishing gradients and overfitting, thereby reducing detection stability in low signal-to-noise ratio environments.

Balancing detection performance, parameter scale, and computational complexity, the 3-layer network achieves extreme lightweighting while maintaining excellent detection accuracy and localization performance. Consequently, all subsequent experiments in this paper adopt the 3-layer network as the optimal architecture for the proposed SpecUNeSt-MLP model.

### 4.4. Comparison with existing methods

SpecUNeSt-MLP was compared with LAD [5], FCN [7], RMP [13], SS-LSTM [24], and SS-CNN [9] under identical experimental conditions. RMP, originally developed for two-dimensional segmentation, was adapted to process one-dimensional PSD vectors and produce frequency-bin-level masks. All models used an 8192-point input and an IoU threshold of 0.8. The proposed SpecUNeSt-MLP adopted the three-layer configuration in this comparison.

#### 4.4.1. Detection and localization performance.

Fig 6 compares the detection probability ($P_{d}$), false-alarm probability ($P_{f}$), and average localization error ($E_{avg}$) over SNRs from −4 to 8 dB. The corresponding numerical results are reported in Tables 1–3. This SNR sweep evaluates robustness under controlled noisy conditions, including the most challenging −4 dB case.

**Table 1:** Detection probability $P_{d}$ under different SNR conditions (IoU threshold = 0.8). Higher values are better.

SNR	LAD	FCN	RMP	SS-LSTM	SS-CNN	Ours
−4 dB	0.3315±0.0000	0.6580±0.0329	0.7795±0.0239	0.5016±0.1568	0.6706±0.0734	0.8317±0.0040
−2 dB	0.4101±0.0000	0.7619±0.0335	0.8518±0.0038	0.6552±0.1261	0.7004±0.0577	0.8516±0.0071
0 dB	0.4026±0.0000	0.8009±0.0372	0.8812±0.0162	0.7378±0.0902	0.7983±0.0404	0.9005±0.0089
2 dB	0.3860±0.0000	0.8541±0.0378	0.9391±0.0094	0.8456±0.0712	0.8515±0.0351	0.8971±0.0158
4 dB	0.5348±0.0000	0.8038±0.0415	0.9059±0.0140	0.7240±0.0779	0.7907±0.0444	0.9311±0.0050
6 dB	0.6793±0.0000	0.8520±0.0240	0.9332±0.0109	0.8649±0.0473	0.8687±0.0175	0.9207±0.0128
8 dB	0.4183±0.0000	0.8361±0.0227	0.9203±0.0266	0.8366±0.0675	0.8396±0.0460	0.9465±0.0122
Average	0.4518	0.7953	0.8873	0.7380	0.7885	**0.8970**

**Table 2:** False‑alarm probability $P_{f}$ under different SNR conditions (IoU threshold = 0.8). Lower values are better.

SNR	LAD	FCN	RMP	SS-LSTM	SS-CNN	Ours
−4 dB	0.6268±0.0000	0.4060±0.0740	0.2260±0.0270	0.4516±0.1238	0.3863±0.0813	0.1689±0.0058
−2 dB	0.5232±0.0000	0.2639±0.0372	0.1460±0.0048	0.2890±0.1003	0.3371±0.0658	0.1363±0.0075
0 dB	0.5716±0.0000	0.2656±0.0374	0.1417±0.0195	0.2221±0.0761	0.2362±0.0437	0.1055±0.0097
2 dB	0.6003±0.0000	0.1965±0.0645	0.0833±0.0113	0.1471±0.0572	0.1842±0.0368	0.0926±0.0091
4 dB	0.4516±0.0000	0.2440±0.0525	0.1093±0.0206	0.2338±0.0586	0.2202±0.0323	0.0869±0.0061
6 dB	0.3035±0.0000	0.2698±0.1082	0.0808±0.0089	0.1341±0.0398	0.1483±0.0233	0.0862±0.0127
8 dB	0.5796±0.0000	0.2916±0.0923	0.1081±0.0345	0.1545±0.0643	0.2145±0.0633	0.0762±0.0166
Average	0.5224	0.2768	0.1279	0.2332	0.2467	**0.1075**

**Table 3:** Average localization error $E_{avg}$ under different SNR conditions (IoU threshold = 0.8). Lower values are better.

SNR	LAD	FCN	RMP	SS-LSTM	SS-CNN	Ours
−4 dB	0.0645±0.0000	0.0579±0.0043	0.0434±0.0018	0.0631±0.0077	0.0590±0.0040	0.0397±0.0008
−2 dB	0.0593±0.0000	0.0483±0.0027	0.0368±0.0008	0.0572±0.0063	0.0520±0.0012	0.0351±0.0010
0 dB	0.0508±0.0000	0.0386±0.0032	0.0257±0.0016	0.0508±0.0073	0.0443±0.0050	0.0254±0.0014
2 dB	0.0503±0.0000	0.0348±0.0032	0.0232±0.0009	0.0390±0.0055	0.0415±0.0046	0.0222±0.0010
4 dB	0.0564±0.0000	0.0407±0.0025	0.0309±0.0010	0.0442±0.0052	0.0467±0.0032	0.0286±0.0015
6 dB	0.0401±0.0000	0.0332±0.0013	0.0215±0.0012	0.0407±0.0080	0.0386±0.0052	0.0220±0.0017
8 dB	0.0563±0.0000	0.0450±0.0024	0.0296±0.0013	0.0512±0.0098	0.0475±0.0044	0.0323±0.0017
Average	0.0540	0.0427	0.0302	0.0494	0.0471	**0.0293**

Across the seven SNR conditions, SpecUNeSt-MLP obtains average values of 0.8970, 0.1075, and 0.0293 for $P_{d}$, $P_{f}$, and $E_{avg}$, respectively. RMP is the strongest competing model, with corresponding averages of 0.8873, 0.1279, and 0.0302. SpecUNeSt-MLP therefore improves the average detection probability by 0.0097 and reduces the average false-alarm probability and localization error by 0.0204 and 0.0008, respectively, relative to RMP. At −4 dB, SpecUNeSt-MLP achieves $P_{d}=0.8317$, $P_{f}=0.1689$, and $E_{avg}=0.0397$. Compared with RMP at this most adverse SNR, these correspond to a 0.0522 absolute increase in $P_{d}$, a 0.0571 reduction in $P_{f}$, and a 0.0037 reduction in $E_{avg}$. The comparatively small standard deviations of SpecUNeSt-MLP also indicate stable performance across repeated runs. Although RMP is marginally better at several individual SNR points, SpecUNeSt-MLP provides the best overall performance averaged across the complete SNR range, with its largest advantage appearing in the low-SNR regime.

#### 4.4.2. Computational efficiency.

To comprehensively evaluate the computational efficiency of the proposed model, we report the parameter count, GFLOPs, memory, and batch-one inference latency in Table 4. GFLOPs were calculated for a single 8192-point input. Latency were measured using the same implementation environment and hardware configuration described in Section 4.1.4. The latency values denote the average inference time per sample at batch size 1.

**Table 4:** Computational-efficiency comparison for an 8192-point input.

Method	Params (M)	GFLOPs	Memory(MB)	Latency (ms/sample)
LAD	0	0.0003	0	0.77
FCN	0.0708	0.2001	0.306	3.00
RMP	1.2057	0.9397	4.65	6.41
SS-LSTM	1.2088	3.2124	4.63	10.85
SS-CNN	0.0802	0.6641	0.311	12.02
SpecUNeSt-MLP	0.2008	0.4268	0.827	8.65

SpecUNeSt-MLP contains 0.2008 M parameters and requires 0.4268 GFLOPs per 8192-point sample. Compared with the strongest accuracy baseline, RMP, it reduces the parameter count and computational cost by approximately 83.3% and 54.6%, respectively. It also requires 86.7% fewer parameters and 86.7% fewer GFLOPs than SS-LSTM. Its measured latency is 8.65 ms per sample, which is lower than those of SS-LSTM and SS-CNN, although higher than those of FCN and RMP. These measurements show that SpecUNeSt-MLP does not minimize every individual resource metric; rather, it achieves a favorable accuracy–efficiency trade-off by substantially improving detection robustness and localization accuracy while retaining a compact parameter count and moderate computational cost.

### 4.5. Impact of training sample proportion

We further investigate the data efficiency of SpecUNeSt-MLP by varying the proportion of training samples: 40% (0.4), 60% (0.6), 80% (0.8), and 100% (1.0) of the full training set, while keeping all other hyperparameters fixed (FFT size = 8192, 3-layer network, IoU threshold = 0.8). The results are summarized in Fig 7 with three subfigures showing Detection Probability ($P_{d}$), False Alarm Rate ($P_{f}$), and Average Localization Error ($E_{avg}$), respectively.

As depicted in Fig 7, the proposed model exhibits strong robustness to reduced training data. When the training proportion increases from 0.4 to 1.0, $P_{d}$(left) improves modestly, while both $P_{f}$(center) and $E_{avg}$(right) decrease gradually. Notably, the performance gap across different training proportions becomes even smaller at higher SNR levels.

These results demonstrate that our model maintains favorable performance even when the number of training samples is reduced. Specifically, SpecUNeSt‑MLP achieves competitive performance using merely 40%–60% of the training samples, which highlights its outstanding data efficiency and generalization ability. Such a feature is especially desirable for practical spectrum sensing scenarios, where acquiring large‑scale labeled data is usually expensive and labor‑intensive.

### 4.6. Impact of IoU threshold

To examine the influence of the Intersection over Union (IoU) threshold on final detection performance, we evaluated four different IoU thresholds: 0.6, 0.7, 0.8, and 0.9, while keeping all other experimental settings fixed (FFT size = 8192, 3-layer network, full training set). The results are presented in Fig 8, which consists of three subfigures for Detection Probability ($P_{d}$), False Alarm Rate ($P_{f}$), and Average Localization Error ($E_{avg}$), respectively.

As shown in Fig 8 from (left) to (right), model performance is highly sensitive to the choice of IoU threshold. Theoretically, a lower IoU threshold yields higher detection probability($P_{d}$) and lower false-alarm probability($P_{f}$),yet introduces larger average localization error ($E_{avg}$) for the detected signal bandwidth. Our experimental results are consistent with this theoretical trend.

As the IoU threshold increases, higher localization accuracy can be obtained at the cost of reduced detection probability and elevated false‑alarm probability. Although IoU = 0.9 delivers the best localization precision, it suffers from severe performance degradation under low‑SNR conditions, where high noise leads to substantial missed detections. Therefore, we select an IoU threshold of 0.8 for our spectrum‑sensing task. This setting maintains reasonably high detection probability and a low false‑alarm rate, while ensuring satisfactory accuracy of the detected signal frequency‑band range. Such accuracy is essential for subsequent effective signal extraction using filters.

### 4.7. Ablation study on key modules

To quantify the contribution of each proposed component, we performed an ablation studies on the core modules of SpecUNeSt-MLP under SNR conditions of -4dB, 0 dB, and 4dB. The base model is a standard 1D U-Net without Split-Attention, FIB or rolling MLP. We then incrementally add the Split-Attention, FIB and Bidirectional Rolling MLP (RMLP) modules for comparison. The results are summarized in Table 5.

**Table 5:** Ablation study of the proposed method under different SNR conditions.

Metric	Methods	SNR		
		−4 dB	0 dB	4 dB
$P_{d}$	Base	0.4177 ± 0.0072	0.5564 ± 0.0097	0.6093 ± 0.0029
	Base+Split-Attention	0.5222 ± 0.0187	0.6300 ± 0.0097	0.6604 ± 0.0100
	Base+FIB	0.5391 ± 0.0105	0.6338 ± 0.0052	0.7106 ± 0.0041
	Base+RMLP	0.8198 ± 0.0132	0.8953 ± 0.0062	0.9277 ± 0.0112
	Ours	**0.8317 ± 0.0040**	**0.9005 ± 0.0089**	**0.9311 ± 0.0050**
$P_{f}$	Base	0.4756 ± 0.0078	0.4661 ± 0.0143	0.3734 ± 0.0037
	Base+Split-Attention	0.3974 ± 0.0388	0.3933 ± 0.0078	0.3567 ± 0.0126
	Base+FIB	0.4662 ± 0.0114	0.4376 ± 0.0035	0.3417 ± 0.0025
	Base+RMLP	0.2023 ± 0.0142	0.1271 ± 0.0048	0.1080 ± 0.0149
	Ours	**0.1689 ± 0.0058**	**0.1055 ± 0.0097**	**0.0869 ± 0.0061**
*E*_avg_	Base	0.0735 ± 0.0008	0.0553 ± 0.0014	0.0561 ± 0.0004
	Base+Split-Attention	0.0680 ± 0.0019	0.0561 ± 0.0008	0.0499 ± 0.0014
	Base+FIB	0.0603 ± 0.0007	0.0446 ± 0.0010	0.0426 ± 0.0009
	Base+RMLP	0.0455 ± 0.0026	0.0313 ± 0.0013	0.0324 ± 0.0010
	Ours	**0.0397 ± 0.0008**	**0.0254 ± 0.0014**	**0.0286 ± 0.0015**

The tabulated results reveal that each individual module contributes evident performance gains across all SNR levels. The FIB module can preliminarily refine feature representation and moderately boost detection accuracy. The Bidirectional RMLP further strengthens the model’s capability to capture long-range frequency dependencies, which significantly elevates $P_{d}$ while cutting down both $P_{f}$ and *E*_avg_. Moreover, introducing the Split-Attention mechanism enhances discriminative learning of inter-channel features and further suppresses false alarms. When Split-Attention, FIB and RMLP are fully integrated into the baseline network, a clear synergistic effect emerges. Our complete model achieves the maximum detection probability, minimal false alarm probability and the lowest average localization error at every tested SNR. These observations verify that the three proposed modules are indispensable for SpecUNeSt-MLP to achieve state-of-the-art spectrum sensing performance.

## 5. Conclusion

In this paper, we proposed SpecUNeSt-MLP, a lightweight one-dimensional deep learning framework for radio signal detection based on power spectral density (PSD). By incorporating split-attention into the U-Net encoder and a bidirectional rolling MLP (RMLP) into the bottleneck, the proposed framework effectively captures both local spectral features and long-range frequency dependencies while maintaining low computational complexity.

Extensive experiments on synthetic wideband spectra demonstrate that SpecUNeSt-MLP consistently outperforms LAD, FCN, SS-CNN, and SS-LSTM across SNRs from –4–8 dB in terms of detection probability, false alarm rate, and average localization error. It also achieves better low-SNR performance than the recent RMP baseline. Ablation and sensitivity studies further verify the effectiveness of the proposed modules and indicate that an FFT size of 8192 and a three-layer network provide a favorable accuracy–efficiency trade-off. The model remains robust with reduced training data and achieves a balanced detection–localization performance at an IoU threshold of 0.8.

SpecUNeSt-MLP contains only 0.2008 M parameters and requires 0.4268 GFLOPs for an 8192-point input. On an NVIDIA Tesla V100 GPU, it achieves an average inference latency of 8.65 ms with a batch size of 1, demonstrating its potential for real-time spectrum sensing on resource-constrained platforms.

Despite these promising results, the current study relies mainly on simulated data, with only 820 real-world RF samples. Future work will focus on large-scale real-world validation, generalization under complex interference environments, and deployment on representative edge platforms. Overall, SpecUNeSt-MLP provides an effective and efficient solution for wideband spectrum sensing and shows strong potential for practical cognitive radio applications.
